# Characterization and functional analysis of two novel thermotolerant α-l-arabinofuranosidases belonging to glycoside hydrolase family 51 from *Thielavia terrestris* and family 62 from *Eupenicillium parvum*

**DOI:** 10.1007/s00253-020-10867-7

**Published:** 2020-09-03

**Authors:** Liangkun Long, Lu Sun, Qunying Lin, Shaojun Ding, Franz J. St John

**Affiliations:** 1 College of Chemical Engineering, Nanjing Forestry University, Nanjing, 210037 China; 2 Institute for Microbial and Biochemical Technology, Forest Products Laboratory, USDA Forest Service, One Gifford Pinchot Drive, Madison, WI 53726 USA; 3 Nanjing Institute for the Comprehensive Utilization of Wild Plants, Nanjing, 211111 China

**Keywords:** α-l-arabinofuranosidase, Thermal stability, Calcium, Synergistic degradation, Filamentous fungi

## Abstract

**Abstract:**

Arabinofuranose substitutions on xylan are known to interfere with enzymatic hydrolysis of this primary hemicellulose. In this work, two novel α-l-arabinofuranosidases (ABFs), TtABF51A from *Thielavia terrestris* and EpABF62C from *Eupenicillium parvum*, were characterized and functionally analyzed. From sequences analyses, TtABF51A and EpABF62C belong to glycoside hydrolase (GH) families 51 and 62, respectively. Recombinant TtABF51A showed high activity on 4-nitrophenyl-α-l-arabinofuranoside (83.39 U/mg), low-viscosity wheat arabinoxylan (WAX, 39.66 U/mg), high-viscosity rye arabinoxylan (RAX, 32.24 U/mg), and sugarbeet arabinan (25.69 U/mg), while EpABF62C preferred to degrade arabinoxylan. For EpABF62C, the rate of hydrolysis of RAX (94.10 U/mg) was 2.1 times that of WAX (45.46 U/mg). The optimal pH and reaction temperature for the two enzymes was between 4.0 and 4.5 and 65 °C, respectively. Calcium played an important role in the thermal stability of EpABF62C. TtABF51A and EpABF62C showed the highest thermal stabilities at pH 4.5 or 5.0, respectively. At their optimal pHs, TtABF51A and EpABF62C retained greater than 80% of their initial activities after incubation at 55 °C for 96 h or 144 h, respectively. ^1^H NMR analysis indicated that the two enzymes selectively removed arabinose linked to C-3 of mono-substituted xylose residues in WAX. Compared with the singular application of the GH10 xylanase EpXYN1 from *E. parvum*, co-digestions of WAX including TtABF51A and/or EpABF62C released 2.49, 3.38, and 4.81 times xylose or 3.38, 1.65, and 2.57 times of xylobiose, respectively. Meanwhile, the amount of arabinose released from WAX by TtABF51A with EpXYN1 was 2.11 times the amount with TtABF51A alone.

**Key points:**

*• Two novel α-l-arabinofuranosidases (ABFs) displayed high thermal stability.*

*• The thermal stability of GH62 family EpABF62C was dependent on calcium.*

*• Buffer pH affects the thermal stability of the two ABFs.*

*• Both ABFs enhance the hydrolysis of WAX by a GH10 xylanase.*

**Electronic supplementary material:**

The online version of this article (10.1007/s00253-020-10867-7) contains supplementary material, which is available to authorized users.

## Introduction

Hemicellulose is the second most abundant polysaccharide in the biosphere and is of interest for bioconversion to green chemicals, fuels, biomaterials, functional foods, and pharmaceuticals (Liu et al. 2016; Zhou et al. 2017). Heteroxylans represent the predominant type of hemicellulose found in hardwoods and agricultural crop biomass and is composed of a backbone of β-1,4-linked xylose residues variably substituted with α-l-arabinofuranose, 4-*O*-methyl-α-d-glucuronic acid, galactose, and acetic acid moieties (Moreira and Filho 2016; Zhou et al. 2017). In the cereal bran xylans, xylose residues may be substituted both with mono- and di-substituted α-l-arabinofuranosyl (Ara*f*) residues at O-2 and/or O-3 positions (McCleary et al. 2015), and the content of arabinose can be as high as 33–45% depending on the source (Saha 2000). Ara*f* residues also exist in arabinan, gum arabic, and arabinogalactan (Saha 2000). The presence of Ara*f* substitutions enhanced the resistance of xylan to enzymatic hydrolysis (Thakur et al. 2019).

Exo-α-l-arabinofuranosidases (ABFs, EC 3.2.1.55) catalyze the hydrolysis of terminal non-reducing α-1,2-, α-1,3-, or α-1,5-linked Ara*f* residues from arabinose-substituted polysaccharides or shorter oligosaccharides (Thakur et al. 2019; Yang et al. 2015). Based on protein sequence similarities, ABFs have been classified into glycoside hydrolase (GH) families 2, 3, 43, 51, 54, and 62 of the Carbohydrate-Active enZYmes (CAZy) database (http://www.cazy.org/) (Lombard et al. 2014). Members of the GH43 family are active on α-1,5-linked l-Ara*f* oligosaccharides or are specific for α-1,2- and or α-1,3-linked Ara*f* from xylan (Mewis et al. 2016), those from GH51 or 54 hydrolyze mono- and di-substituted Ara*f* side chains on arabinoxylan or arabinan (dos Santos et al. 2018), and GH62 family ABFs seem to be specialized in removing mono-substituted Ara*f* residues from arabinoxylans (Wilkens et al. 2017). ABFs of families GH2, 3, 51, and 54 act on the glycosidic linkage by a retaining mechanism, and members of GH43 and 62 families (clan GH-F) display a five-bladed propeller arrangement with an inverting mechanism of hydrolysis (Maehara et al. 2014; Numan and Bhosle 2006; Wang et al. 2014).

Literature indicates that ABFs are one of the rate-limiting enzymes in xylan degradation (Saha 2000), and their application showed a strong synergistic role with endo-xylanase in degradation of arabinoxylans into arabinose, xylose, and xylooligosaccharides (XOS) (Goncalves et al. 2012; Jia et al. 2016; Ravn et al. 2018). Compared with the individual enzymes, the total amount of released sugar from wheat arabinoxylan was increased to 2.92-fold with simultaneous addition of a GH51 ABF and the GH10 endoxylanase XynBE18 (Yang et al. 2015). Currently, diverse ABFs from both fungal and bacterial sources have been reported, which may provide important information for the understanding of the enzymes (Amore et al. 2012; Bouraoui et al. 2016; Hu et al. 2018; Kaur et al. 2015; Shinozaki et al. 2015; Wilkens et al. 2017; Yang et al. 2015).

Thermotolerant biomass-degrading enzymes usually perform well due to their higher stability and potential for enzyme recycling (Brunecky et al. 2018; Chadha et al. 2019; de Cassia et al. 2015). In addition, elevation of hydrolysis temperature is beneficial to dissolution of substrates and products, enhancement of mass transformation, and reduced risk of microbial contamination (Berka et al. 2011). To date, the reported thermostable ABFs were mainly from thermophilic bacteria. For example, several GH51 family ABFs with high catalytic temperatures (over 60 °C) and high thermal stability were isolated from *Thermobacillus xylanilyticus*, *Thermotoga petrophila*, or *Paenibacillus* sp. DG-2 (Debeche et al. 2000; dos Santos et al. 2011; Lee and Lee 2014). Thermophilic fungi and some mesophilic fungi are important producers of thermotolerant enzymes. *Thielavia terrestris*, which encodes numerous hemicellulolytic enzymes, has a maximum growth temperature over 50 °C (Berka et al. 2011). The mesophilic fungus *Eupenicillium parvum* produces diverse hydrolytic enzymes with high temperature optimum and high thermal stabilities, e.g., endoglucanase, β-glucosidase, and xylanase (Long et al. 2016). In the present study, two thermotolerant ABFs belonging to the fungi *T. terrestris* (TtABF51A) and *E. parvum* (EpABF62C) and belonging to GH families 51 or 62, respectively, were characterized. The two ABFs were functionally compared regarding the liberation of Ara*f* from different substrates and degradation of wheat arabinoxylan individually, together with a GH10 endoxylanase from *E. parvum*.

## Materials and methods

### Strain, media, and culture conditions

The filamentous fungus *E. parvum* 4-14 (CCTCC M2015404) (Long et al. 2016) was maintained on potato dextrose agar (PDA) slant in our laboratory. *Escherichia coli* strain Tran10 (TransGen, Beijing, China), *Pichia pastoris* GS115 strain (Invitrogen, Carlsbad, CA*,* USA), and *Trichoderma reesei* D-86271 (VTT, Espoo, Finland) were used for plasmid propagation or protein expression, respectively. Mandels’ medium was prepared according to the previously reported method (Long et al. 2016) and used for fermentation of *T. reesei*. YPD, BMGY, and BMMY medium were prepared according to the Pichia Expression Kit Instruction Manual (Invitrogen). The recombinant *P. pastoris* strain (named Pp-xyn1) containing the gene *EpXyn*1 from *E. parvum* 4-14 was used to express endo-β-1,4-xylanase EpXYN1 of GH 10 family (Long et al. 2018b).

### Gene synthesis, protein expression, and purification of TtABF51A

A putative GH51 ABF encoding gene from *T. terrestris* NRRL 8126 (TtABF51A) was synthesized according to the published sequence (GenBank XP_003649438.1) and cloned into vector plasmid pPICZαA by the Genscript Biotech Corporation (Nanjing, China). To facilitate purification, 6× his tag was included at the C-terminal of the expressed protein. To express the gene in *T. reesei*, the gene fragment without the signal sequence was PCR amplified using primers Abf51A_f2 and Abf51A_r2 (Supplementary Table S1) and the gene fragment was ligated into plasmid pAg-PTcbh1 (Long et al. 2018c) by Hieff Clone™ One Step PCR Cloning Kit (Yeasen, Shanghai, China). The recombinant plasmid was introduced into *T. reesei* strain D-86271 by *Agrobacterium tumefaciens–*mediated transformation method (Long et al. 2018a; Mullins et al. 2001). Fungal transformants were selected on PDA plates containing 100 μg/mL of hygromycin B, identified by PCR amplification with gene-specific primers, and then preserved on PDA slant tubes. The plasmid pAg-PTcbh1 was employed as a control in the transformation experiment.

For production of protein TtABF51A, the randomly selected fungal transformants were cultured on PDA plates for sporulation. About 1 × 10^8^ fungal spores were inoculated in a 250-mL flask containing 50 mL of Mandels’ medium with 1% glucose as carbon source, and grew at 200 rpm and 28 °C for 2 days on a shaker. Five milliliters of liquid inoculant was transferred into a 2-L flask containing 300 mL of Mandels’ medium with 1% (*w*/*v*) lactose as carbon resource and 60 μL antifoam 204 (Sigma, St. Louis, MO, USA), and incubated at 160 rpm and 27 °C for 5 days. During the fermentation, an appropriate amount of NaOH solution (1 M) was added into the culture broth to maintain the pH value at 4.5–5.0. The crude extract of protein TtABF51A was concentrated by ultrafiltration through a 10-kDa cut-off membrane (Amicon 8400; Millipore, Billerica, MA, USA). Then, immobilized metal affinity chromatography (IMAC) was performed by applying the crude protein to Chelating Sepharose Fast Flow (Amersham Biosciences, Uppsala, Sweden) in the Ni^2+^ form. The protein TtABF51A was eluted with elution buffer (pH 7.4) containing 20 mM Tris–HCl, 500 mM NaCl, and 300 mM imidazole. After desalting by ultrafiltration (5-kDa cut-off membrane), the protein was further purified by a BioLogic Duo-Flow medium-pressure chromatography system (Bio-Rad, Hercules, CA, USA) equipped with a HiLoad Superdex 200 column, using the gel filtration buffer (GFB) consisting of 25 mM Tris and 150 mM NaCl, pH 7.5. This buffer was subsequently used for enzyme storage. After concentration by ultrafiltration, the pure protein was quantified by absorption at *OD*_280nm_ with a NanoDrop 2000 (Thermo Fisher, Carlsbad, CA, USA) and concentration determined according to the molar extinction coefficient, and stored at − 80 °C.

### Cloning, protein expression, and purification of EpABF62C

A gene encoding a putative ABF which was classified as a GH family 62 (EpABF62C) was identified in the *E. parvum* genome by BLAST analysis of fungal transcriptome data (unpublished data). The cDNA fragment of the protein was isolated from the fungus by RT-PCR amplification with specific primers Abf62C_f1 and Abf62C_r1 (Supplementary Table S1). Fungal culture, total RNA extraction, and first-strand cDNA synthesis were conducted by previous methods (Long et al. 2018b). After cloning into the vector pEASY-Blunt (Transgene, Beijing, China), the target gene fragment was sequenced by GENEWIZ Biotech. Co. Ltd. (Suzhou, China).

To express the protein in *P. pastoris* cells, the gene was re-amplified with primers Abf62C_f2 and Abf62C_r2, and *Eco*RI and *Xba*I restriction fragments were ligated into plasmid pPICZɑA. The recombinant plasmid pPIC-GH62C was linearized with enzyme *Sac*I and introduced into *P. pastoris* GS115 by electroporation with a Gene Pulser II Electroporation System (Bio-Rad, Hercules, CA, USA). Yeast transformants were selected on YPD plates supplemented with 100 μg/mL of Zeocin (Invitrogen). Protein expression was conducted in BMMY medium using 0.8% methanol as inducer according to the Pichia Expression Kit Instruction Manual (Invitrogen, Carlsbad, CA, USA). Target protein was purified from fermentation supernatant by the same method as described earlier for TtABF51A and analyzed by SDS-PAGE.

### Activity assay of recombinant protein

Different substrates including 4-nitrophenyl-α-l-arabinofuranoside (pNPAra*f*) (Sigma, St. Louis, MO, USA), low-viscosity wheat arabinoxylan (WAX), high-viscosity rye arabinoxylan (RAX), and sugarbeet arabinan (SBA) (Megazyme, Bray, Ireland) were used to detect the activities of recombinant ABFs. The reaction system consisted of sodium acetate buffer (0.1 M, pH 4.5 or 5.0) 50 μL, pNPAra*f* (20 mM) 5 μL, enzyme (20–50 ng/μL) 10 μL, and dH_2_O 35 μL. The mixture was incubated at 70 °C for 10 min, and 400 μL of NaCO_3_ (0.2 M) was added. The released nitrophenol was detected by measuring the absorbance at *OD*_405nm_ and calculating the concentration with a standard curve. For the natural substrates, the reaction mixture contained 40 μL of the same buffer, 50 μL of substrate (10 mg/mL), and 10 μL of enzyme (25 ng/μL). Reaction was performed at 65 or 70 °C for 20 min, and stopped by incubating at 99 °C for 10 min. The released reducing sugar was quantified by Somogyi–Nelson method (Nelson 1944). Reaction mixtures were diluted 2-fold with addition of 100 μL of dH_2_O and mixed with 200 μL of the Somogyi reagent. After incubation at 99 °C for 20 min, 200 μL of the arsenomolybdate color reagent was mixed with the cooled mixture. This was then diluted with 2 mL of dH_2_O, mixed, and the absorbance at *OD*_540nm_ measured using a spectrophotometer (Genesys™ 10S UV-Vis; Thermo Scientific, Waltham, MA, USA). The amount of reducing sugar was calculated according to arabinose standard curve. One unit of ABF activity was defined as the amount of enzyme required to release 1 μmol of arabinose or 4-nitrophenyl from the corresponding substrate per minute under the reaction conditions.

### TLC analysis of hydrolysis product

Thin-layer chromatography (TLC) was employed to analyze the hydrolysis products of natural substrates by the recombinant enzymes. In a 1.5-mL tube, 50 μL of substrate (10 mg/mL) was mixed with 10 μL of enzyme (50 ng/μL) and 40 μL of NaAC buffer (0.1 M, pH 4.5). The mixtures were incubated at 60 °C for 20 min or 4 h. For every sample, 4 μL of hydrolysis product was spotted onto a TLC plate (Alltech, Lexington, KY, USA) and the plates were developed with the solvent system chloroform/acetic acid/water (18:21:3, *v*/*v*/*v*) with two ascensions (St John et al. 2006). The TLC plates were air dried for 30 min and visualized by spraying with a methanol containing 3% H_2_SO_4_ and 6.5 mM *N*-(1-naphthyl) ethylendiamine dihydrochloride followed by heating at 110 °C for 10 min (Bounias 1980). Arabinose and xylose were used as standards.

### Effect of temperature or pH on the enzymatic activity and stability

The optimum catalysis conditions of recombinant proteins on different substrates were determined in the buffers with different pH values or under different temperatures in the same buffer with optimized pH. The reaction buffers included glycine–HCl buffer (0.1 M, pH 2.0–3.5), sodium acetate buffer (0.1 M, pH 3.5–6.0), sodium phosphate buffer (pH 6.0–8.0), and glycine–NaOH buffer (pH 8.0–11.0). Relative activity was calculated using the maximum activity which was assayed in the same type of buffer as 100%.

The pH stability of the enzymes was tested by incubating pure protein (0.1 μg/μL) in different buffers (pH 2.0–11.0) at 4 °C for 24 h, and the residual enzymatic activities were detected under standard conditions. To evaluate the thermal stabilities of recombinant enzymes, pure proteins (0.1 μg/μL) were mixed with different buffers (pH 2.0–11.0) and incubated at 55 °C for 24 h, or mixed with 50 mM of sodium acetate buffer (pH 4.5 or 5.0) and treated under different temperatures up to 1 week. The residual enzymatic activities were assayed using the method described previously. Relative activity (%) was calculated using the activity of untreated enzyme as 100%.

### Effect of metal ions on the activity and stability of recombinant enzymes

Different metal ions including Mg^2+^ (MgCl_2_), Ca^2+^ (CaCl_2_), Co^2+^ (CoCl_2_), Ni^2+^ (NiSO_4_), Fe^2+^ (FeSO_4_), Mn^2+^ (MnSO_4_), Zn^2+^ (ZnSO_4_), Cu^2+^ (CuSO_4_), or EDTA (sodium salt) were added into the reaction system at the final concentration of 1 or 5 mM, respectively. Then, the activities of recombinant enzymes were assayed under the standard conditions. Relative activity (%) was calculated using the activity of untreated enzyme as 100%.

Meanwhile, pure enzymes were treated with EDTA solution to remove divalent metal ions. Purified protein (250–300 μL) was transferred into a Slide-A-Lyzer G2 dialysis cassette (Thermo Scientific) and dialyzed against 50 mM of EDTA in the GFB storage buffer for 20 h, and dialyzed in the same buffer without EDTA for 24 h (changed the buffer every 8 h) at 4 °C. The treated protein (0.1 μg/μL) was incubated with divalent metal ions at a final concentration of 1 mM, and subjected to determination of activity or thermal stability as described earlier, using untreated proteins as controls.

### Substrate specificity and kinetic parameters

Specific activities of recombinant proteins on different substrates were measured under optimal conditions. To determine the kinetic parameters of the enzymes, the activities of target proteins were assayed under standard conditions with a substrate concentration response curve. With these data, the *K*_m_ and *V*_max_ values were calculated by the software GraphPad Prism 7.04 using nonlinear regression (https://www.graphpad.com/).

### ^1^H NMR analysis of modes of actions of ABFs toward arabinoxylan

The modes of actions of TtABF51A and EpABF62C toward wheat arabinoxylan were evaluated using one-dimensional ^1^H nuclear magnetic resonance (NMR) spectra as previously described (Wang et al. 2014; Sarch et al. 2019). In a 2-mL tube, 300 μL of WAX (20 mg/mL) was mixed with 10 μL of TtGH51A or EpGH62C (0.3 μg/μL) and 290 μL of NaAC buffer (0.1 M, pH 4.5). After incubation at 55 °C for 24 h, the enzymatic reactions were quenched by boiling for 10 min. Following each enzyme treatment, the mixtures were precipitated by addition of ethanol to approximately 66% with incubation at 4 °C for 3 h and then separated by centrifugation at 15,000×*g* for 10 min. The pellet was suspended in 0.6 mL of ddH_2_O and again precipitated with ethanol by the same method. To solubilize the xylan that precipitated upon releasing the L-Ara*f* substituents by the enzyme treatment, the pellet was suspended in 0.6 mL of 10 mM NaAC buffer (pH 5.0) and digested by 2 μg of the endoxylanase EpXYN3 of GH11 family (Long et al. 2018b) at 45 °C for 2 days. The reaction mixtures were treated at 100 °C for 10 min and then lyophilized and dissolved in 0.6 mL of D_2_O two times. ^1^H NMR spectra were obtained at 25 °C in 5.0-mm NMR tubes (Norell) by using an AVANCE III HD 600-MHz spectrometer with a scan number of 16 and a relaxation delay of 10 s. The data were recorded by using Topspin 3.5 (Bruker, Shanghai, China) and analyzed with MestReNova 14.0.0 software (Mestrelab Research, Escondido, CA) as described previously (Wang et al. 2014; Sarch et al. 2019). At the same time, the ratios of mono- and di-substituted α-1,2- or α-1,3-l-Ara*f* on WAX and RAX were compared by using the same ^1^H NMR analysis, except that the substrates were not treated with the ABFs.

### Synergistic degradation of arabinoxylan with ABFs and xylanase

Xylanase EpXYN1 was prepared by fermentation of *P. pastoris* strain Pp-xyn1 according to the previous method (Long et al. 2018b). In a 2-mL centrifuge tube, 50 μL of substrate (10 mg/mL) and 100 μL of sodium acetate solution (100 mM, pH 4.5) were mixed with individual enzyme or combinative enzymes (0.5 μg of each enzyme), and increased the reaction volume to 200 μL by the addition of distilled water. The reaction mixture was incubated in a water bath with 55 °C for 24 h, and the released reducing sugars were quantified by Somogyi–Nelson test (Nelson 1944) or HPLC analysis. For HPLC analysis, the reactions were precipitated with 80% ethanol on ice for 40 min to remove the remaining undigested polysaccharide. After centrifugation at 9000×*g* for 10 min, the supernatant was dried using a Savant SpeedVac® System (Thermo Scientific) and re-dissolved in ultra-pure water. The released xylose, arabinose, and xylooligosaccharide were quantified by an Agilent Infinity HPLC system (Agilent Technologies, Santa Clara, CA, USA) equipped with a SHODEX Sugar SH 1821 column (SHODEX, Shanghai, China) with 0.01 N of H_2_SO_4_ as eluent at a flow rate of 0.8 mL/min and a column temperature of 60 °C. The degree of synergy is defined as the ratio of the total amount of liberated sugars resulting from the combined enzyme treatment to the sum of the sugars released by the xylanase or ABFs used separately (Yang et al. 2015).

### Bioinformatic analysis

The amino acid sequences of target proteins were subjected to the online BLAST analysis (https://blast.ncbi.nlm.nih.gov/Blast.cgi). Prediction of signal peptide, theoretical molecular weight (MW), and p*I* were conducted by SignalP-5.0 (http://www.cbs.dtu.dk/services/SignalP/) or ProtParam tool (https://web.expasy.org/protparam/), respectively. After removing signal sequences, the two protein sequences with their close relatives from GenBank were aligned using ClustalX2 (Larkin et al. 2007), and a phylogenetic tree was constructed using the neighbor-joining method with the Poisson model and 1000-bootstrap test iterations using Mega 7 software (Kumar et al. 2018). The three-dimensional (3D) model structures of TtABF51A and EpABF62C were predicted using the online I-TASSER server (https://zhanglab.ccmb.med.umich.edu/I-TASSER/) (Yang and Zhang 2015).

## Results

### Molecular characteristics of TtABF51A and EpABF62C

**Fig. 1:**
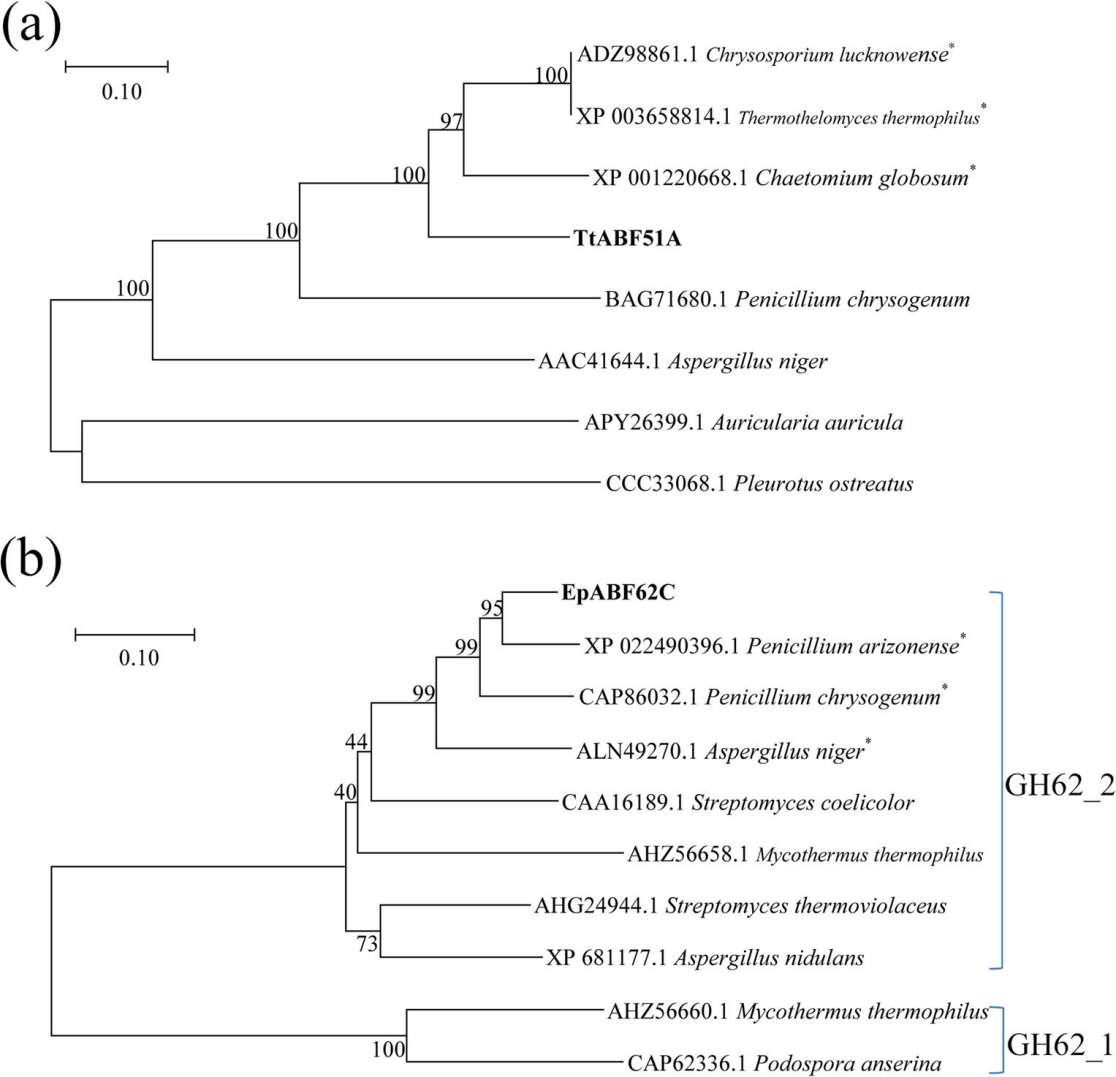
Phylogenetic tree analysis of proteins TtABF51A (**a**) and EpABF62C (**b**) with the arabinofuranosidases belonging to GH51 or GH62 families. Mature protein sequences were used to construct the phylogenetic tree. The trees were constructed with MEGA 7.0 using neighbor-joining method under the Poisson model. Numbers on branches indicate bootstrap values from 1000 replications. * indicates uncharacterized protein

The full length of cDNA fragments encoding the proteins TtABF51A (640 amino acids, aa) and EpABF62C (328 aa) were 1920 and 984 bp (GenBank MN855573), respectively. A 20 aa or 26 aa signal peptide was predicted at the N terminus of protein TtABF51A or EpABF62C, respectively. The N terminus of TtABF51A contains a predicted carbohydrate-binding module (CBM) (aa positions 56–192) belonging to CBM family 4_9. As determined using the ProtParam tool, the theoretical MW and p*I* were 67.8 kDa and 5.71 for TtABF51A, or 32.8 kDa and 5.89 for EpABF62C. TtABF51A has its greatest homology to an uncharacterized GH51 protein (GenBank XP_003658814.4) from *Thermothelomyces thermophilus* with approximately 71% identity and shares 53% identity with a characterized GH51 ABF (GenBank BAG71680.1) from *Penicillium chrysogenum* (Sakamoto et al. 2013; Shinozaki et al. 2015). EpABF62C showed 73% identity with a characterized ABF (GenBank CAA16189) belonging to GH62 from the bacterium *Streptomyces coelicolor* (Maehara et al. 2014). The constructed phylogenetic trees indicated that TtABF51A and EpABF62C were affiliated with the ABFs belonging to GH51 or 62 family (62_2 subfamily), respectively (Fig. 1a, b).

Conserved residues of ABFs belonging to GH51 (e.g., catalytic residues Glu356 and Glu432) or GH62 (e.g., “SHG” motif) families were found in the sequences of TtABF51A or EpABF62C, respectively (Supplementary Fig. S1). The predicted protein structure of TtABF51A consists of a CBM domain, a (β/α)_8_-barrel, and a β-sandwich domain (Supplementary Fig. S2) with an overall structural fold similar to the dual-domain glycoside hydrolase families 30, 39, and 44 (St John et al. 2010), and has a high structural similarity (I-TASSER TM score 0.707) to the structure of the family GH51 ABF (PDB 1qw9A) from *Geobacillus stearothermophilus* T-6 (Hovel et al. 2003). In the predicted structure model of EpABF62C, the common 5-fold β-propeller structure and the conservative catalytic triad (amino acid residues D54, D162, and E214) were observed (Supplementary Fig. S2). The structure is highly similar (TM score 0.993) to the structure of an ABF of GH62 family (PDB 5ubjA) from *Aspergillus nidulans* (Contesini et al. 2017).

### Expression and purification of recombinant proteins

**Fig. 2:**
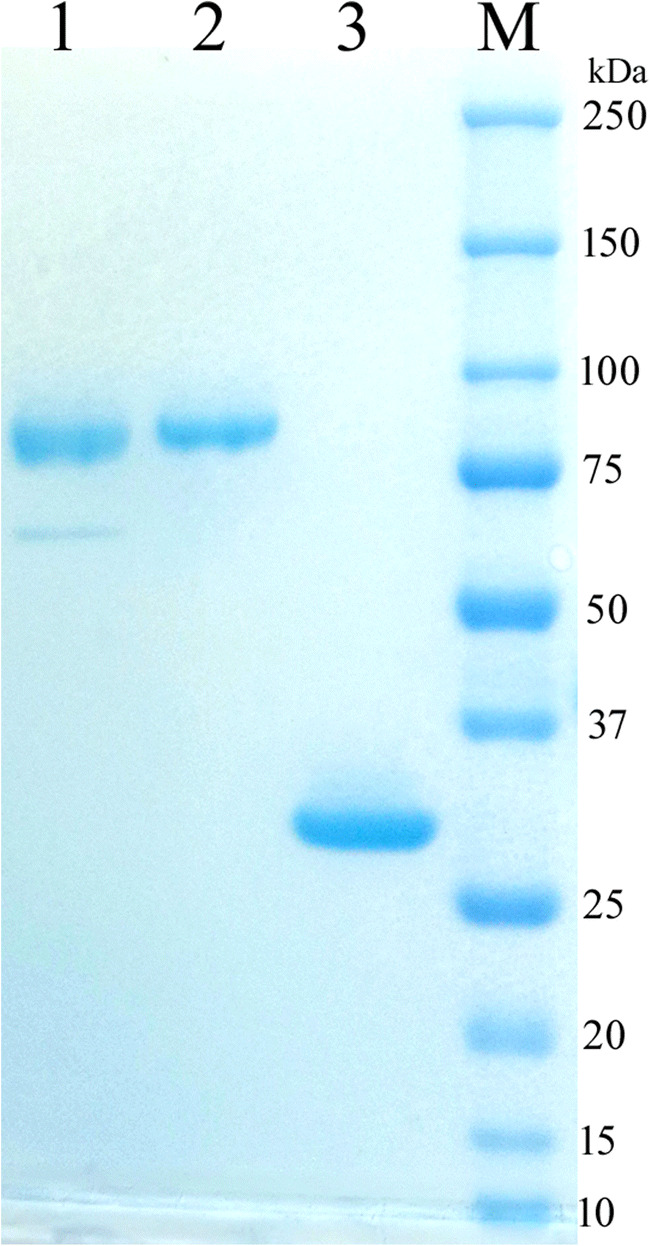
Analysis of recombinant proteins by SDS-PAGE. *Lane 1*, protein TtABF51A following purification by Ni^2+^ IMAC; *lane 2*, protein TtABF51A after second purification with chromatography system; *lane 3*, pure EpABF62C following Ni^2+^ IMAC; *M*, protein marker. About 6–8 μg of protein was loaded in each lane for electrophoresis on a 4–15% gradient SDS-PAGE gel

Proteins TtABF51A and EpABF62C were successfully produced by fermentation of the recombinant *T. reesei* or *P. pastoris* strains, respectively. Under the expression conditions, 13.5 μg/mL of TtABF51A and 149.4 μg/mL of EpABF62C were purified from the crude fermentation broths. On the SDS-PAGE gel, the apparent MW of recombinant TtABF51A was close to 80 kDa, which is higher than the theoretical value (68.6 kDa). This difference may be due to three N-glycosylated positions (N46, N207, and N516) that were predicted in the mature protein of TtABF51A by the online analysis (http://www.cbs.dtu.dk/services/NetNGlyc/). For EpABF62C, the apparent MW by SDS-PAGE was in agreement with the theoretical MW value (Fig. 2).

### Biochemical characterization of recombinant proteins

#### Hydrolytic product and optimal catalytic conditions

**Fig. 3:**
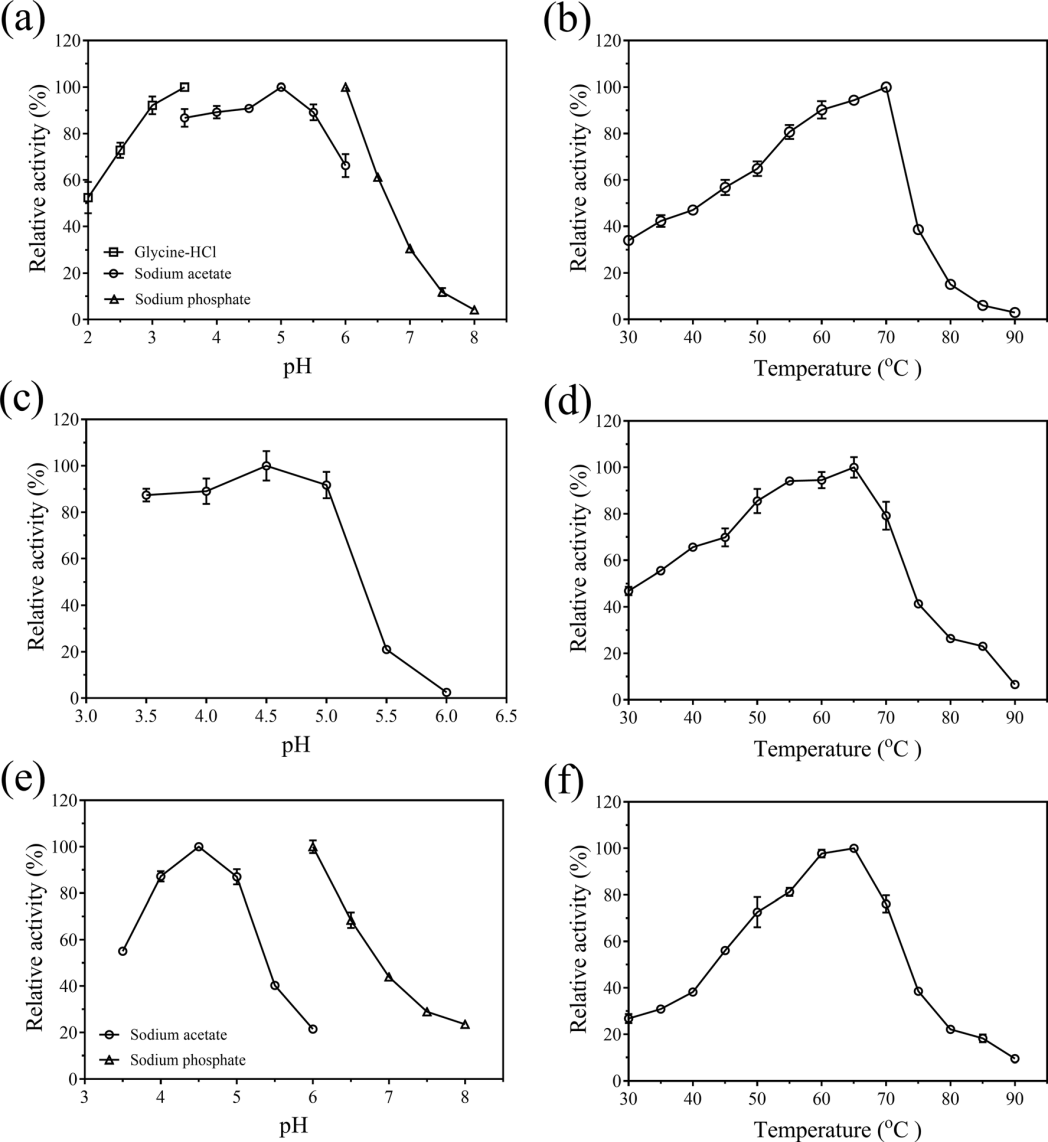
Optimal pHs or temperatures of the recombinant enzymes on synthetic or natural substrates. **a**, **b** Effect of pH or temperature on the activity of enzyme TtABF51A toward pNPAra*f*. **c**, **d** Effect of pH or temperature on the activity of enzyme TtABF51A toward wheat arabinoxylan. **e**, **f** Effect of pH or temperature on the activity of enzyme EpABF62C toward wheat arabinoxylan. Except as indicated, enzymatic activities were assayed at 70 °C (**a**) or 65 °C (**c**, **e**), and in sodium acetate buffer with pH 5.0 (**b**) or 4.5 (**d**, **f**). Relative activity was calculated using the maximum activity as 100%. Error bars represent SDs from three independent assays

TLC analysis indicated that only arabinose was released from WAX, RAX, or SBA by the two proteins, and TtABF51A showed higher activity than EpABF62C on SBA (Supplementary Fig. S3). In hydrolysis of pNPAra*f*, TtABF51A showed the highest activity at pH 5.0 and 70 °C (Fig. 3a, b) while the optimal hydrolysis conditions for the natural substrate WAX was at pH 4.5 and 65 °C (Fig. 3c, d). Using RAX or SBA as substrate, TtABF51A displayed the best activity at pH 4.0 and 65 °C, respectively (Supplementary Fig. S4a–d). The best pH and temperature for the activity of EpABF62C was 4.5 and 65 °C toward WAX (Fig. 3e, f) or RAX (Supplementary Fig. S4e and f).

**Fig. 4:**
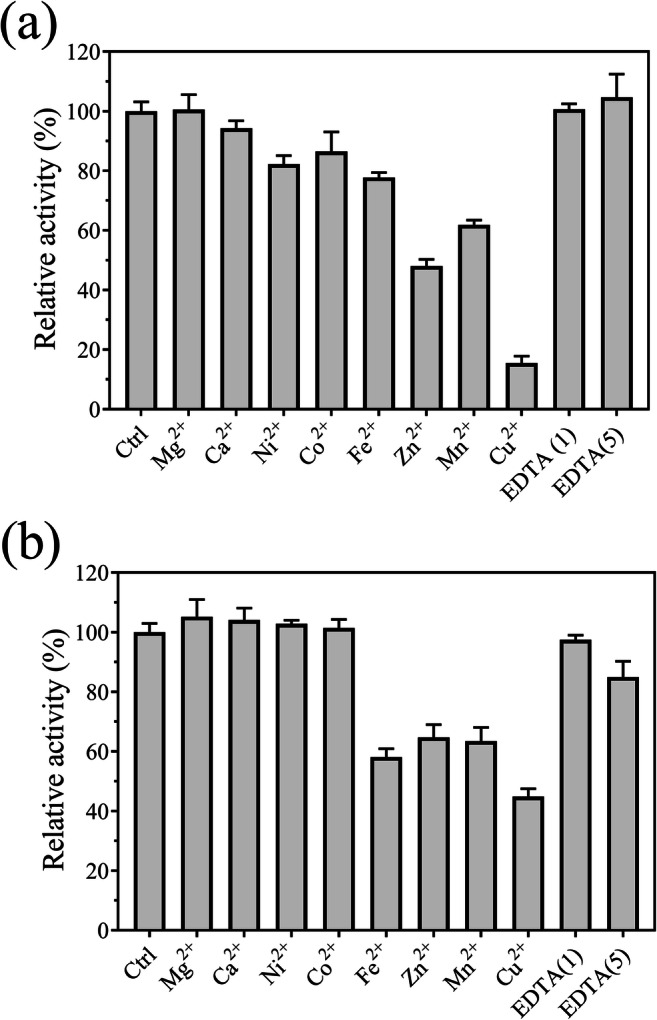
Effect of metal ions on the activities of recombinant enzymes TtABF51A (**a**) or EpABF62C (**b**). Each metal ion or EDTA was added into the reaction buffer at final concentration of 1 or 5 mM (only EDTA). Enzymatic activities on WAX were assayed under standard conditions. Ctrl, untreated enzyme. Relative activity was calculated using the activity of the untreated enzyme as 100%. Error bars represent SDs from three independent assays

Effect of metal ions on the enzymatic activities and thermal stabilities:

The activity of TtABF51A was inhibited by Cu^2+^, Zn^2+^, Mn^2+^, Fe^2+^, Co^2+^, and Ni^2+^ to various degrees (Fig. 4a). This enzyme lost 52 or 85% activity in the presence of Zn^2+^ or Cu^2+^, respectively. Among these metal ions, Cu^2+^, Zn^2+^, Mn^2+^, and Fe^2+^ showed inhibitory effect on EpABF62C. The addition of Mg^2+^, Co^2+^, or Ni^2+^ did not change the enzymatic activity. Meanwhile, EDTA did not change the activity of TtABF51A; however, EpAbf62C activity decreased 15% in the presence of 5 mM EDTA (Fig. 4b).

**Fig. 5:**
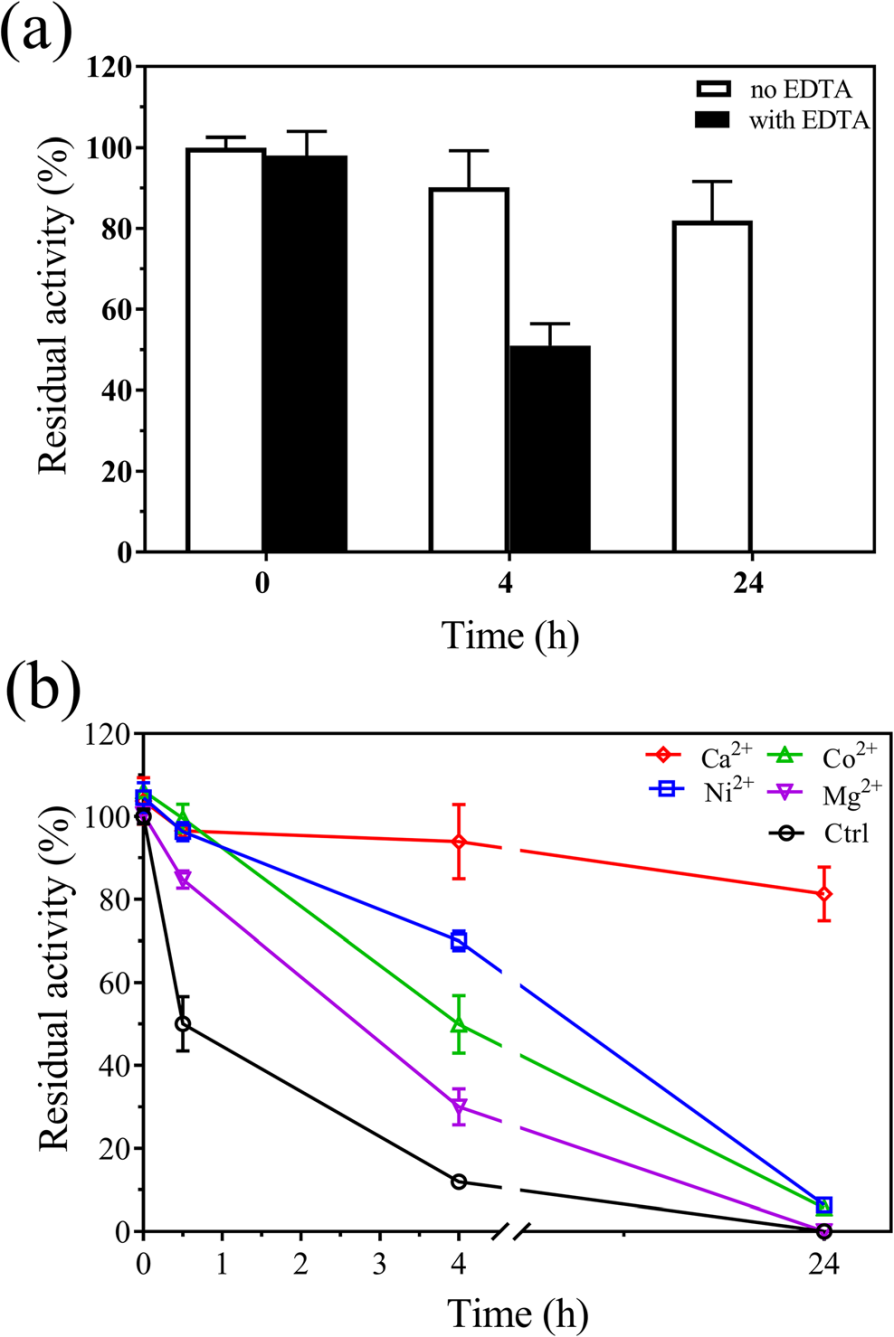
Effect of metal ions on the thermal stability of recombinant enzyme EpABF62C. **a** Analysis of thermal stability of enzyme EpABF62C with or without EDTA. Pure protein EpABF62C (0.1 μg/μL, 100 μL) was mixed with EDTA (final concentration 1 mM) at 4 °C for 1 h. The residual activity of the enzyme was assayed after incubation at 55 °C for 4 or 24 h, respectively. **b** Comparison of the thermal stabilities of enzyme EpABF62C in the presence of different metal ions. Enzyme EpABF62C without metal ions was prepared by the EDTA treatment as described in the “Materials and methods” section and mixed with different metal ions (final concentration 1 mM) at 4 °C for 1 h. After treatment at 60 °C for 0.5 to 24 h, the residual activity of the enzyme was determined under standard conditions using WAX as substrate. Ctrl, no metal ions. Relative activity was calculated using the initial activity of the untreated enzyme as 100%. Error bars represent SDs from three independent assays

Continued incubation of EpABF62C in 1 mM of EDTA at 55 °C led to eventual loss of detectable activity (Fig. 5a). In a separate study, EDTA was used under mild conditions to remove excess metal ions, and Mg^2+^, Co^2+^, Ni^2+^, or Ca^2+^ were separately tested to determine which has a role in protein stability. EpABF62C was incubated at 60 °C for 0.5 to 24 h in the presence of the additional metal ions. After 0.5 h of incubation, the enzyme maintained high (over 84%) activity for all the cation treatments. During extended incubation times, the activity of EpABF62C remained high only in the presence of calcium ion (Fig. 5b). These results indicated that calcium plays a critical role in the thermal stability of EpABF62C.

#### Effects of pH on thermal stability of the recombinant enzymes

**Fig. 6:**
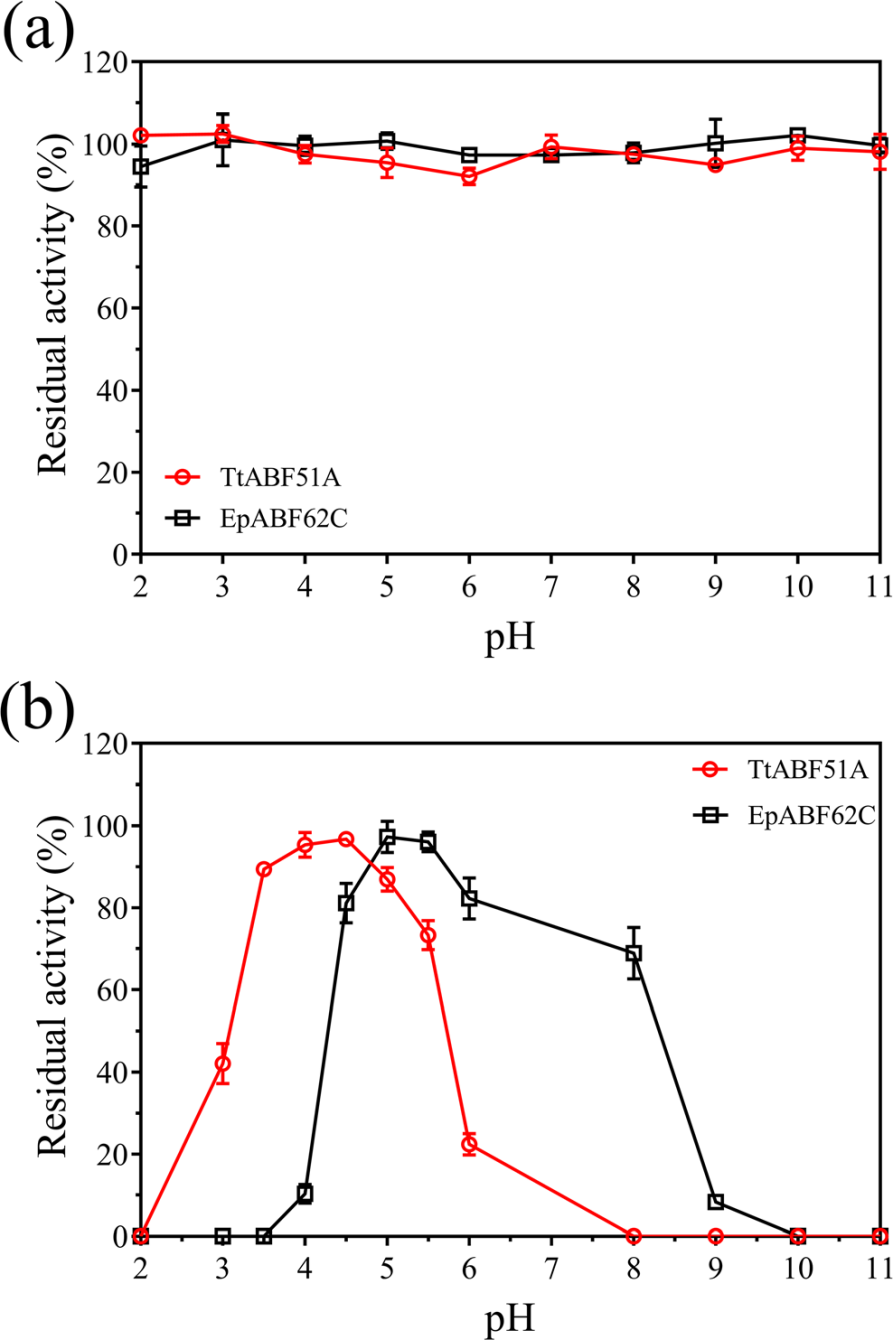
Effect of pH on the stabilities of recombinant enzymes under low (**a**) or high (**b**) temperatures. Pure enzymes (0.1 μg/μL) were added in glycine–HCl buffer (pH 2.0–3.0), sodium acetate buffer (pH 4.0–6.0), sodium phosphate buffer (pH 7.0), or glycine–NaOH buffer (pH 8.0–11.0), respectively. After incubated at 4 °C (**a**) or 55 °C (**b**) for 24 h, residual activities of the enzymes were assayed under standard conditions using WAX as substrate. Because the proteins precipitated in the buffer after treatment at 55 °C, the data of pH 7.0 in Fig. 7b were removed. Relative activity was calculated using the activity of the untreated enzyme as 100%. Error bars represent SDs from three independent assays

Both TtABF51A and EpABF62C maintained over 90% of their activities following incubation in buffers from pH 2.0 to 11.0 at 4 °C for 24 h (Fig. 6a). A similar study performed at 55 °C resulted in activity loss of both enzymes with reaction optimal pH divergence. For TtABF51A, the enzyme kept the highest (over 90%) level of activity in the buffers with pH 4.0 or 4.5, and treatment with pH 2.0 or over 8.0 led to the loss of the full activity of the enzyme. EpABF62C maintained maximum activity after treatment at pH 5.0 or 5.5, and quickly decreased the residual activity with higher or lower pH conditions (Fig. 6b). The thermal stabilities of the two enzymes were strongly affected by environmental pH value.

**Fig. 7:**
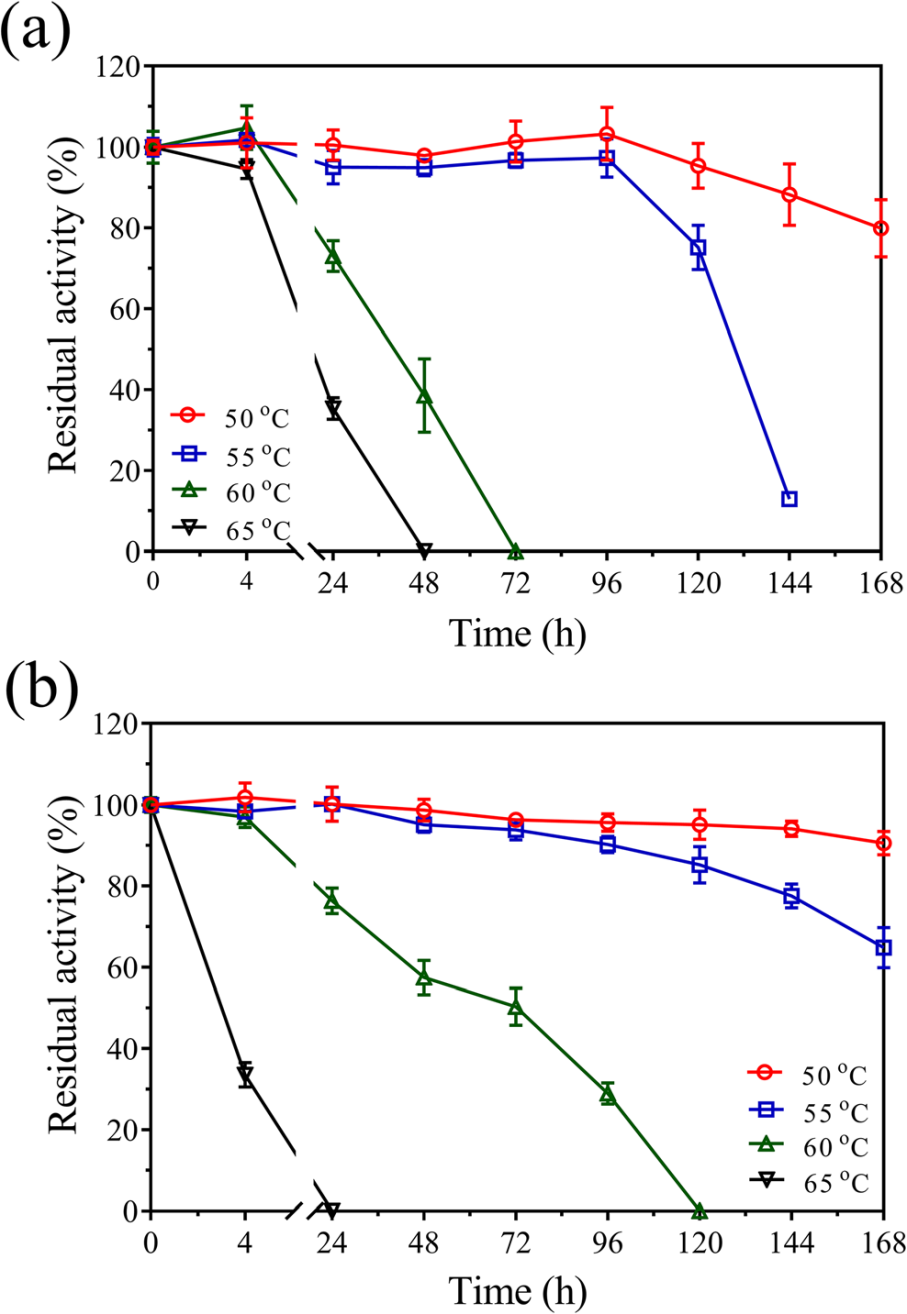
Thermal stabilities of recombinant enzymes TtABF51A (**a**) and EpABF62C (**b**). Pure enzymes (final concentration 0.1 μg/μL) were added in 50 mM of sodium acetate buffer with pH 4.5 (for TtABF51A) or 5.0 (for EpABF62C), and incubated at 50, 55, or 60 °C for up to 168 h. Enzyme EpABF62C was treated with 5 mM of CaCl_2_ before the thermal stability test. After 4-fold dilution, residual activities of the enzymes on WAX were assayed under standard conditions. Relative activity was calculated using the activity of the untreated enzyme as 100%. Error bars represent SDs from three independent assays

The thermal stabilities of the two enzymes were further evaluated under their respective optimum protein stability pH condition over time at 50, 55, 60, and 65 °C, respectively. Both TtABF51A and EpAbf62C maintained 80 and 90% of their respective activities following 168 h of incubation at 50 °C. Both enzymes also performed well at 55 °C with TtABF51A maintaining over 80% of its initial activity following 96 h and EpABF62C following 144 h. At 60 or 65 °C, the two enzymes lost their activities relatively quickly (Fig. 7a, b).

#### Specific activities and kinetic parameters

The kinetic parameters of recombinant enzymes toward different substrates were calculated according to the generated substrate concentration kinetic curves (Table 1, Supplementary Fig. S5). As can be seen from Table 1, TtABF51A showed broad activity on the tested substrates including pNPAra*f* (83.39 U/mg), WAX (39.66 U/mg), RAX (32.24 U/mg), and SBA (25.69 U/mg). The enzyme displayed a higher substrate affinity (lower *K*_m_) on RAX rather than on WAX. At a low substrate concentration (below 4 mg/mL), TtABF51A showed a higher activity on RAX than that on WAX (Supplementary Fig. S6). Enzyme EpABF62C displayed high activity on RAX (94.10 U/mg) and WAX (45.46 U/mg) but not pNPAra*f* or arabinans. Calcium slightly affected the specific activity and kinetic parameters of the enzyme while using WAX as substrate.

**Table 1:** Specific activity and kinetic constants of recombinant enzymes toward various substrates

Enzyme	Substrate	Specific activity (U/mg)	*K*_m_ (mM or mg/mL)^d^	*V*_max_ (μmol/min/mg)	*k*_cat_ (s^−1^)
TtABF51A	pNPAra*f*	83.39 ± 3.10	0.30 ± 0.02	107.80 ± 2.54	123.31 ± 2.91
	WAX	39.66 ± 0.93	4.63 ± 0.22	63.24 ± 1.35	72.34 ± 1.54
	RAX	32.24 ± 0.28	1.77 ± 0.12	39.17 ± 0.70	44.81 ± 0.80
	SBA	25.69 ± 1.16	5.52 ± 0.40	36.61 ± 1.03	41.88 ± 1.18
EpABF62C	pNPAra*f*	0.26 ± 0.02^c^	–	–	–
	WAX^a^	42.58 ± 2.28	4.98 ± 0.53	69.93 ± 2.89	39.46 ± 1.63
	WAX^b^	45.46 ± 2.44	6.08 ± 0.70	74.50 ± 3.64	42.04 ± 2.05
	RAX^b^	94.10 ± 3.11	7.73 ± 0.85	178.70 ± 8.75	100.84 ± 4.94
	SBA^b^	1.93 ± 0.08^c^	–	–	–

Except as indicated, all of the enzymatic activities were assayed under standard conditions. The specific activities toward natural substrates were assayed using 8 mg/mL of substrate. The *k*_cat_ values of TtABF51A and EpABF62C were calculated based on the theoretical MW values 68.63 and 33.86 kDa, respectively. ^a^Ca^2+^ was removed from the enzyme; ^b^the enzyme was treated with CaCl_2_ (2 mM); ^c^the reaction time was 1 h; ^d^the unit of *K*_m_ for pNPAra*f* is mM

pNPAra*f*, 4-nitrophenyl-α-l-arabinofuranoside; WAX, wheat arabinoxylan (low viscosity); RAX, rye arabinoxylan; SBA, sugarbeet arabinan. ND, not detectable; –, no analysis

### Regioselectivity of ABFs toward arabinoxylan

**Fig. 8:**
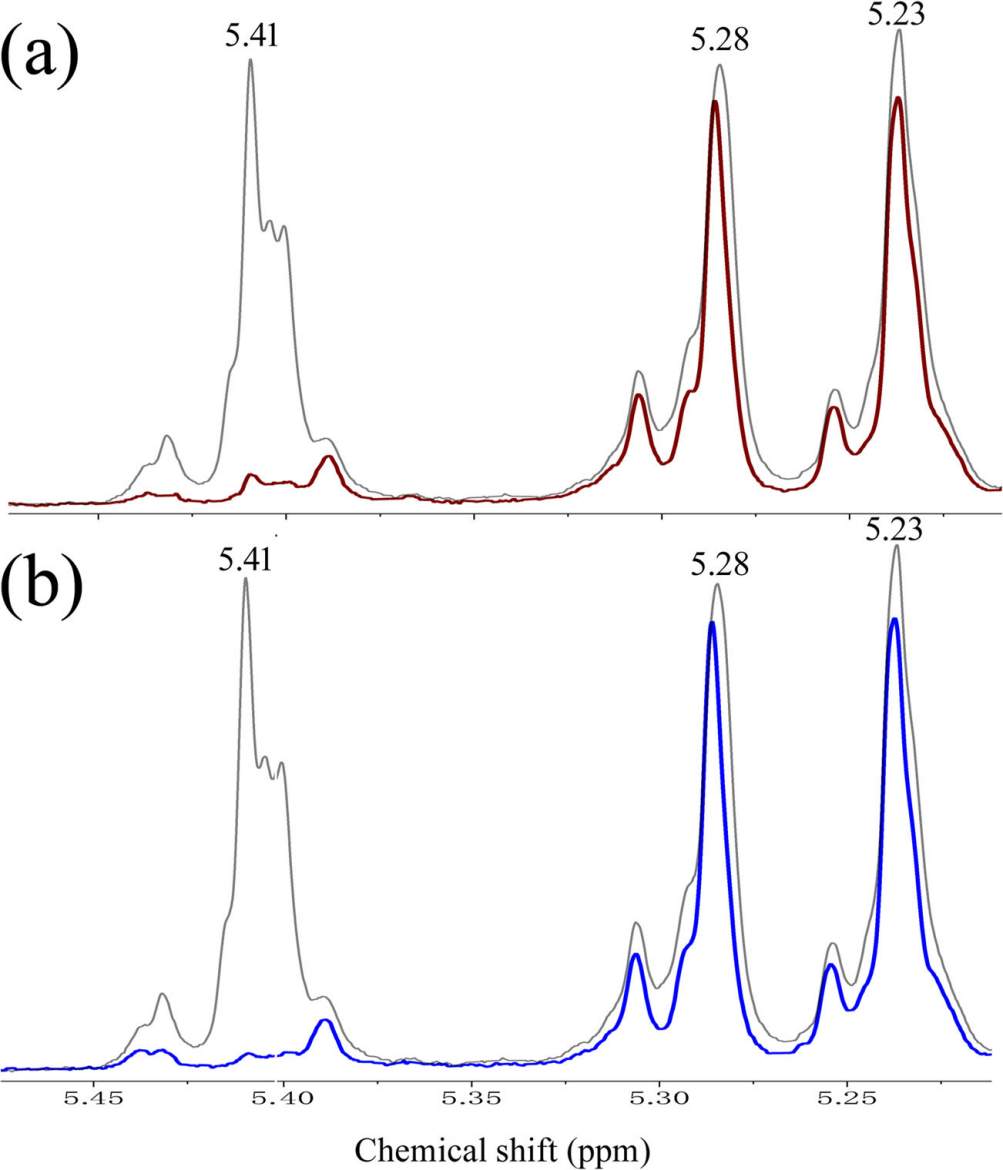
^1^H NMR analysis of enzymatic products of wheat arabinoxylan. **a** Treatment of low-viscosity wheat arabinoxylan (WAX) with (red line) or without (gray line) enzyme TtABF51A. **b** Treatment of WAX with (blue line) or without (gray line) enzyme EpABF62C. The peaks with chemical shifts at 5.41, 5.28, and 5.23 ppm represent the mono-substituted α-1,3-l-Ara*f*, di-substituted α-1,3-l-Ara*f*, and di-substituted α-1,2-l-Ara*f* in the substrate, respectively

^1^H NMR analysis indicated that WAX contains three types of α-l-Ara*f* substitutions with the corresponding signal, including α-1,3-l-Ara*f* linked to C-3 of mono-substituted xylose, and α-1,3-l-Ara*f* and α-1,2-l-Ara*f* linked to C-3 and C-2 of di-substituted xylose with chemical shifts at 5.41, 5.28, and 5.23 ppm, respectively (Fig. 8). The signals of chemical shifts were in agreement with the literature data (Pitkanen et al. 2009; Sakamoto et al. 2011; Wang et al. 2014). Treatment with TtABF51A or EpAbf62C resulted in the disappearance of the signal at 5.41 ppm but not the signals at 5.28 and 5.23 ppm (Fig. 8a, b), indicating the two enzymes selectively acted on the singly substituted α-1,3-l-Ara*f* in the arabinoxylan. This NMR approach was further utilized to confirm previously reported differences in the nature of Ara*f* substitution between WAX and RAX. The substrate RAX contains the same three types of α-l-Ara*f* substitutions, but the relative intensity (%) of α-1,3-l-Ara*f* in this arabinoxylan was about two times of that in WAX (Supplementary Fig. S7). RAX was not used for the regioselectivity analysis of TtABF51A or EpAbf62C due to the severe precipitation of the substrate after treating with the two ABFs.

### Improvement of WAX degradation by synergistic role of xylanase and ABFs

The hydrolytic products of WAX by the individual or the combined enzymes were quantified by HPLC analysis (Table 2). Only arabinose was liberated from the substrate under the action of enzymes TtABF51A or EpABF62C. The amount of released xylose or xylobiose from WAX by the combination of TtABF51A and EpXYN1 was 2.49 times or 3.38 times of that by EpXYN1 alone, respectively. Meanwhile, the amount of released arabinose from WAX by the combined enzymes was 2.11 times that of TtABF51A alone. Compared with the individual enzymes, the combined enzymes EpXYN1 and EpABF62C released 3.38 times of xylose or 1.65 times of xylobiose from the substrate, respectively. The amount of released arabinose did not change by this enzyme combination. When the both ABFs were combined with EpXYN1, the amounts of liberated xylose and xylobiose were up to 4.81 times and 2.57 times of those by single xylanase, respectively. The degrees of synergy were 2.07, 1.16, and 1.98 for the combinations of EpXYN1 with TtABF51A, or EpABF62C, or the two ABFs, respectively. These results from HPLC analysis were in agreement with reducing-end analysis by the Somogyi–Nelson method (Supplementary Fig. S8).

**Table 2:** Hydrolytic products of wheat arabinoxylan by different enzyme combinations

Enzyme combination	Xylose (mg/g)	Xylobiose (mg/g)	Xylotriose (mg/g)	Arabinose (mg/g)	Degree of synergy
EpXYN1	24.26 ± 0.55	92.54 ± 2.01	73.69 ± 1.76	ND	–
TtABF51A	ND	ND	ND	87.54 ± 2.56	–
EpABF62C	ND	ND	ND	81.74 ± 0.99	–
TtABF51A + EpABF62C	ND	ND	ND	87.21 ± 4.72	–
EpXYN1 + TtABF51A	60.39 ± 3.45	313.00 ± 18.48	17.63 ± 3.16	185.07 ± 8.66	2.07
EpXYN1 + EpABF62C	81.91 ± 3.36	152.29 ± 6.86	ND	82.92 ± 4.31	1.16
EpXYN1 + TtABF51A + EpABF62C	116.60 ± 6.63	238.14 ± 2.79	ND	196.00 ± 3.36	1.98

In 200 μL of sodium acetate buffer (50 mM, pH 4.5), 0.5 mg of wheat arabinoxylan (low viscosity) was mixed with xylanase (EpXYN1) and/or arabinofuranosidases (TtABF51A or EpABF62C). The dosage of each enzyme was 0.5 μg per reaction, and the same mass of bovine serum albumin was used as a control. The amount of monosaccharides and oligosaccharides were quantified by HPLC analysis

ND, not detectable; −, no data

## Discussion

The thermophilic fungus *T. terrestris* and the mesophilic fungus *E. parvum* are important sources for production of thermotolerant enzymes (Garcia-Huante et al. 2017; Long et al. 2016; Tang et al. 2019). In the present study, two ABF (TtABF51A and EpABF62C) encoding genes were obtained from the two fungi by artificially synthesis or RT-PCR method. On the basis of sequence analysis, proteins TtABF51A (640 aa) and EpABF62C (328 aa) were classified as GH51 and GH62 families, respectively. A predicted CBM domain (147 aa) belonging to CBM_4_9 superfamily exists at the N-terminal of TtABF51A. There is only limited documentation of the CBM of the GH51 family ABFs. Sin et al. (2016) reported that the GH51 ABF FaARA1 from *Fragaria × ananassa* contains a CBM corresponding to the superfamily CBM_4_9, and the recombinant CBM protein showed a strong affinity to homogalacturonans and a low affinity to microcrystalline cellulose. Due to the various roles of CBMs in polysaccharide degrading enzymes (Gilbert et al. 2013; Guillen et al. 2010), further research is needed to clarify the CBM function of TtABF51A. Proteins TtABF51A and EpABF62C have medium (53%) or high (73%) sequence identities with other characterized ABFs, respectively*.* The two proteins contain conserved residues, e.g., for the active sites and/or the “SHG” motif of ABFs belonging to GH51 or GH62 families. The predicted structure of the catalytic region of TtABF51A was organized into a (β/α)_8_-barrel domain and a β-sandwich domain, which is consistent with the crystal structures of other GH51 ABFs from *G. stearothermophilus* T-6 or *Clostridium thermocellum* (Hovel et al. 2003; Taylor et al. 2006). Meanwhile, the typical 5-fold β-propeller structure and the conserved catalytic triad of ABFs of the GH62 family (Contesini et al. 2017; Maehara et al. 2014; Wang et al. 2014) were predicted in the protein model structure of EpABF62C.

TtABF51A and EpABF62C displayed the highest catalytic activities at high temperature (65 °C) and acidic pH (4.0–5.0) toward natural substrates. TtABF51A showed considerable activity against different substrates including pNPAra*f*, arabinoxylans (from wheat or rye), and arabinan (from sugarbeet). Like most of ABFs from GH62 family (Sarch et al. 2019; Wang et al. 2014; Wilkens et al. 2017), EpABF62C showed specificity for the release of arabinose from arabinoxylans. Like some ABFs of the GH43 family (Ahmed et al. 2013; Valls et al. 2016), EpABF62C displayed about two times greater specific activities toward RAX than those on WAX. A reasonable explanation was that the difference of arabinose-substituted xylose residues distributed in the two substrates (Ahmed et al. 2013; Valls et al. 2016). In this work, it was confirmed by NMR that the substrate RAX contains approximately two times greater α-1,3-l-Ara*f* mono-substituted xylose than WAX, as described in the previous research (Pitkanen et al. 2009). Furthermore, EpABF62C showed specificity in removing mono-substituted α-1,3-l-Ara*f* residues from WAX by ^1^HNMR analysis. The regioselectivity of EpABF62C toward the substrate and the different content of mono-substituted α-1,3-l-Ara*f* between the two substrates resulted in the enzyme having a higher specific activity against RAX than against WAX_._ TtABF51A showed the same regioselectivity toward the arabinoxylan and only displayed a higher activity on RAX than that on WAX at a low substrate concentration (below 4 mg/mL). It was speculated that the activity of the enzyme was affected by the high viscosity of the substrate under the assay conditions.

The catalytic activities of ABFs have been shown to be affected by some divalent metal ions (Hu et al. 2018; Kaur et al. 2015; Komeno et al. 2019). In our study, the activity of TtABF51A was suppressed by Cu^2+^, Zn^2+^, Mn^2+^, Fe^2+^, Ni^2+^, Co^2+^, and Ca^2+^ at different levels. Meanwhile, cations Cu^2+^, Fe^2+^, Zn^2+^, and Mn^2+^ negatively affected the activity of EpABF62C. In literature, a calcium ion is normally observed in the central channel of 5-fold β-propeller structures of ABFs belonging to GH62 or GH43 families (Contesini et al. 2017; Kaur et al. 2015; Santos et al. 2014; Wang et al. 2014). Several ABFs of the GH43 family are reported to need Ca^2+^ for enzymatic activity (Ahmed et al. 2013; de Camargo et al. 2018). The specific activity of EpABF62C had a slight change in the presence of calcium, which is consistent to the result that Ca^2+^ did not significantly affect the catalytic activities of ABFs of the GH62 family (Kaur et al. 2015; Siguier et al. 2014; Wang et al. 2014). The addition of EDTA (5 mM) led to a decrease of the activity of EpABF62C under the tested conditions. Furthermore, our data indicated that calcium was a critical factor for the thermal stability of the enzyme. The conservative “SHG” motif of GH62 family (Wilkens et al. 2017) involved in Ca^2+^ coordination exists in the protein sequence of EpABF62C. Ahmed et al. (2013) found that Ca^2+^ ion imparted the thermal stability of enzyme Ct43Araf from *C. thermocellum* using protein melting-curve analysis. Calcium-dependent thermal stability was reported in diverse proteins including xylanase and glucosidase (Kobayashi et al. 2011; Shi et al. 2013). Part of the explanation was that calcium binding led to structural stability of CMB domain or (β*/*α)_8_-barrel in these proteins (Ahmed et al. 2013; de Sanctis et al. 2010; Kobayashi et al. 2011; Shi et al. 2013).

The two enzymes exhibited high stabilities in a wide pH region (2.0–11.0) at a low temperature (4 °C) but not at a high temperature (55 °C). The thermal stabilities of the enzymes at 55 °C were tightly related to the pH value of buffer. Enzyme TtABF51A showed the highest thermal stability at pH 4.0–4.5, which overlaps the optimal pH for catalytic ability. For EpABF62C, pH 5.0 was the best for the thermal stability and high (85–90%) catalytic activity. Under such reaction pH conditions, long hydrolysis times or recyclable utilization of enzymes may be considered. Under the optimal conditions, enzymes TtABF51A and EpABF62C were stable after incubation at 55 °C for 4 or 6 days, respectively. The two enzymes displayed higher thermal stabilities than many reported ABFs (Hu et al. 2018; Maehara et al. 2014; Sarch et al. 2019; Tu et al. 2019; Wang et al. 2014; Zheng et al. 2018).

Previous studies have indicated that debranching is a key step in the conversion of hemicellulose into monosaccharides or shorter oligosaccharides (Gao et al. 2011; Moreira and Filho 2016; Zheng et al. 2018). Endo-xylanase EpXYN1 (GH10 family) is a thermotolerant enzyme from *E. parvum* and can hydrolyze different xylans into xylobiose, xylotriose, and xylose (Long et al. 2018b). Compared with individual enzymes, the amounts of released xylose or xylobiose from WAX were increased 0.6–3.8 times by EpXYN1 in combination with TtABF51A and/or EpABF62C. Obviously, the removal of Ara*f* residues on the xylose residues enhanced the hydrolytic action of EpXYN1. Similar to what has been previously reported (Hu et al. 2018; Yang et al. 2015), the hydrolysis action of EpXYN1 together with TtABF51A increased the release of arabinose from WAX by 1.11 times. The same phenomenon did not appear in the synergistic degradation of EpXYN1 and EpABF62C. Combined with the same regioselectivities of the two enzymes toward the arabinose polysaccharides WAX, a possible speculation is that TtABF51A can remove the α-1,3-l-Ara*f* or α-1,2-l-Ara*f* from di-substituted xylose residues in arabinose oligosaccharides. In addition, the highest level of xylose but not xylobiose (or xylotriose) was released from WAX by EpXYN1 in combination with the two ABFs. More studies need to be done to understand the detailed mode of action of the two enzymes toward different substrates and explain the mechanism of synergistic degradation.

## Electronic supplementary material

ESM 1 (PDF 1062 kb)
