# Supplementary material for: Characterization and functional analysis of two novel thermotolerant α-l-arabinofuranosidases belonging to glycoside hydrolase family 51 from Thielavia terrestris and family 62 from Eupenicillium parvum
Source: Appl Microbiol Biotechnol. 2020 Sep 3;104(20):8719–33. doi: 10.1007/s00253-020-10867-7 (PMC7502447; doi:10.1007/s00253-020-10867-7)
Supplement: Supplementary file 1 — (PDF 1062 kb) [file 253_2020_10867_MOESM1_ESM.pdf]

Applied Microbiology and Biotechnology

Characterization and functional analysis of two novel thermotolerant α-L-arabinofuranosidases belonging to glycoside hydrolase family 51 from *Thielavia terrestris* and family 62 from *Eupenicillium parvum*

Liangkun Long[1,2#], ORCID：0000-0001-7302-6330

Lu Sun[1#], ORCID：0000-0001-8763-8901

Qunying Lin[3], ORCID：0000-0002-5134-8002

Shaojun Ding[1]*, ORCID：0000-0002-8359-9252

Franz J. St. John[2]*, ORCID：0000-0003-3458-5628

[1] College of Chemical Engineering, Nanjing Forestry University, Nanjing, 210037, China;

[2] Institute for Microbial and Biochemical Technology, Forest Products Laboratory, USDA Forest Service, Madison, WI, 53726, USA;

[3] Nanjing Institute for the Comprehensive Utilization of Wild Plants, Nanjing, 211111, China.

[#] These authors contributed equally to this work.

* Corresponding author:

Franz J. St John

Institute for Microbial and Biochemical Technology, Forest Products Laboratory, USDA Forest Service, One Gifford Pinchot Drive, Madison, WI, USA, 53726;

E-mail: fjstjohn@gmail.com

Shaojun Ding,

College of Chemical Engineering, Nanjing Forestry University, Nanjing, Jiangsu 210037, China

Tel: +86 25 85427939

Fax: +86 25 85418873

E-mail: dshaojun@hotmail.com

Supplementary Materials

Table S1 The information of oligonucleotides

Primer	Sequence (from 5′ to 3′)[a]
Abf51A_f2	ttggccacagctcgtgctcaggtgacgctgtctgtcgcgaagt
Abf51A_r2	ctttcgcacggagctctcgaggagtttttgttctagaaagctggc
Abf62C_f1	attcaaaccatcgcttgaccaa
Abf62C_r1	catctaccgccgcacatct
Abf62C_f2	ccggaattcgactgcgcacttccgtcgact
Abf62C_r2[b]	ctagtctagatca**gtgatggtgatggtgatggtgatg**attcttcagggtaagcacac

[a] The underline nucleotide sequences indicate restriction enzyme sites; [b] Histidine-tag (the bold nucleotide sequences) is contained in the primer

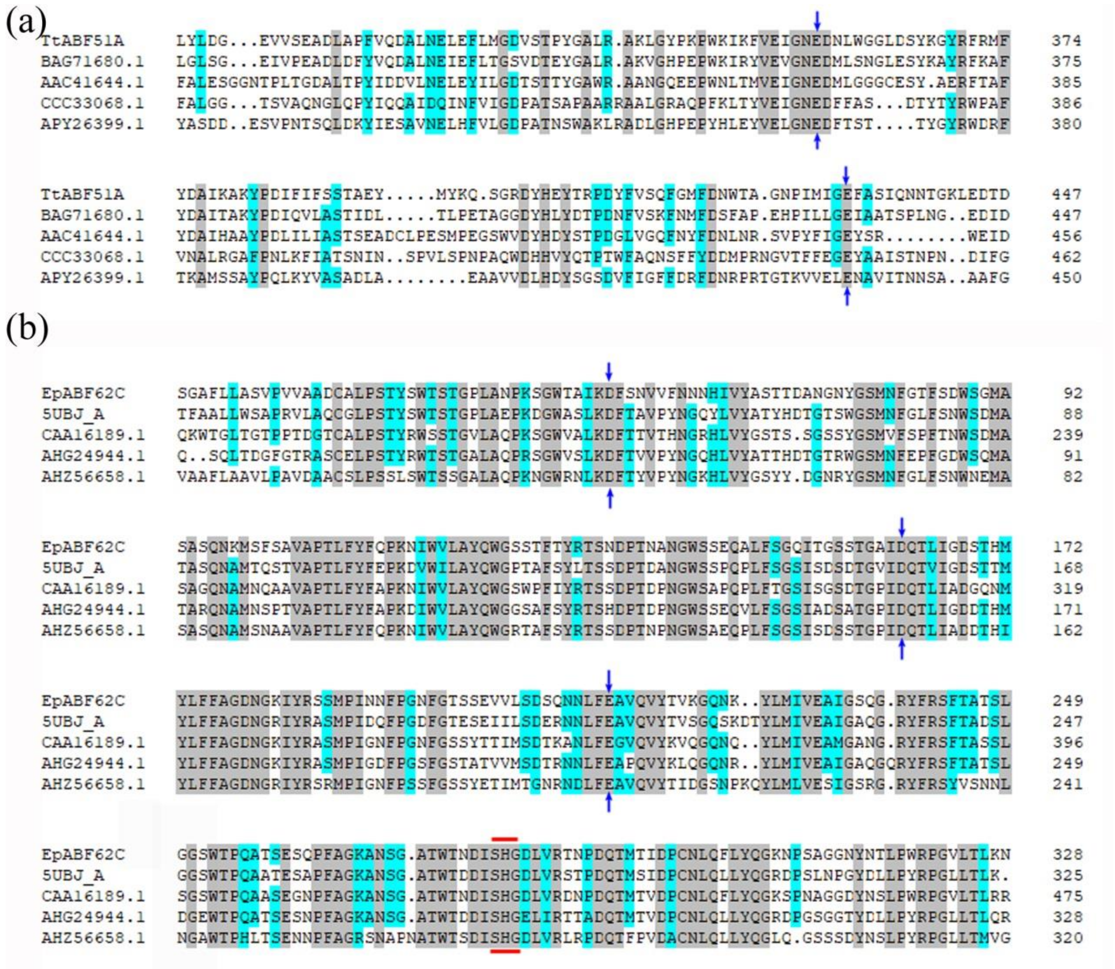

Figure **S1** Sequence alignment of proteins TtABF51A (**a**) or EpABF62C (**b**) with the related arabinofuranosidases. The accession numbers BAG71680.1, AAC41644.1, CCC33068.1, APY26399.1 indicated the ABFs of GH51 family from *P. chrysogenum*, *A. niger*, *P. ostreatus*, and *A. auricula*, respectively. The GenBank accession numbers 5UBJ_A, CAA16189.1, AHG24944.1, AHZ56658.1 indicated the ABFs of GH62 family from *A. nidulans*, *S. coelicolor*, *S. thermoviolaceus*, *M. thermophilus*, respectively. Sequences alignments were conducted by the ClustalX2 and edited with the DNAMAN version 10. The predicted conserve residues (active sites) were rendered as blue arrows, and the domain "SHG" in the GH 62 family was marked by red lines.

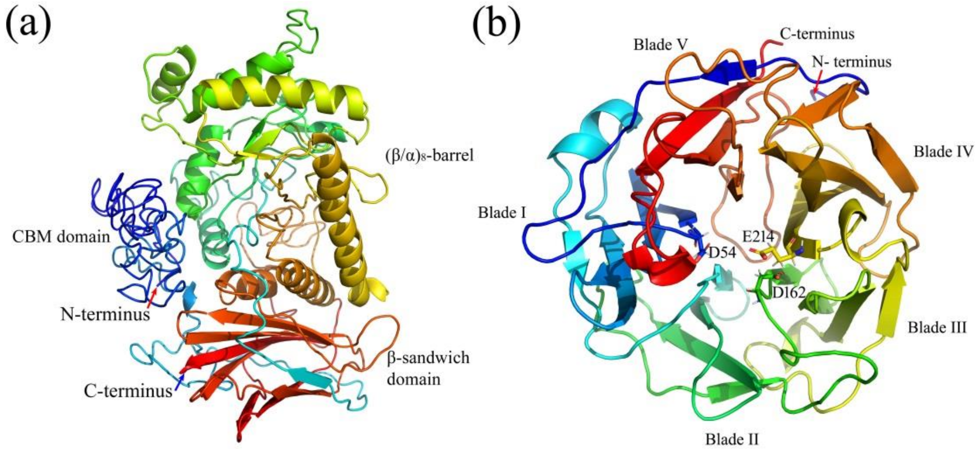

Figure S2 Predicted 3-D structures of proteins TtABF51A (**a**) and EpABF62C (**b**). The predicted structure of TtABF51A consists of a CBM domain, a $(\beta/\alpha)_8$-barrel and a β-sandwich domain. Five-bladed β-propeller fold (blades I to V) and the catalytic triad (D54, D162 and E214) were predicted in the structure of EpABF62C. The three-dimensional model structures were predicted by the I-TASSER server. The C-score and the estimated TM-score are -2.36 and 0.44±0.14 for TtABF51A, or 1.74 and 0.96±0.05 for EpABF62C, respectively.

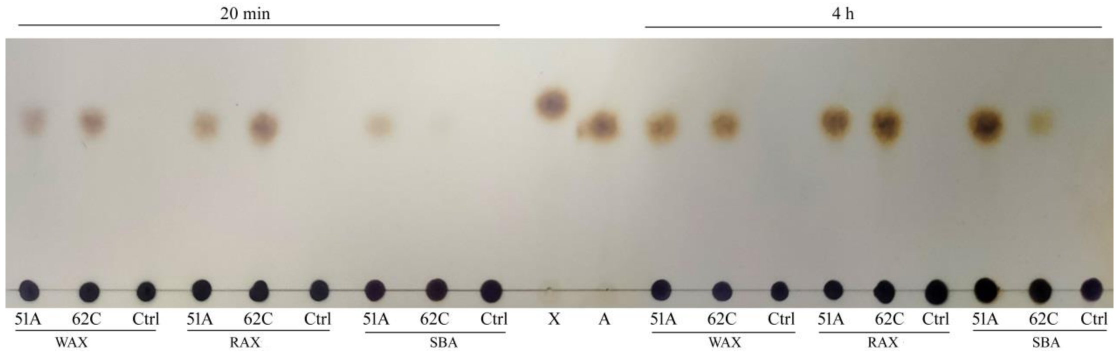

Figure S3 TLC analysis of TtABF51A (51A) or EpABF62C (62C) hydrolysis reactions toward different substrates. In a 1.5-mL tube, 50 μL of substrate (10 mg/mL) was mixed with 10 μL of enzyme (50 ng/μL) and 40 μL of NaAC buffer (0.1 M, pH 4.5). The mixtures were incubated at 60 °C for 20 minutes or 4 hours. For each sample, 4 μL of reaction products was used for TLC analysis. WAX, low viscosity wheat arabinoxylan; RAX, high viscosity rye arabinoxylan; SBA, sugar beet arabinan; X, xylose; A, arabinose; Ctrl, no enzyme.

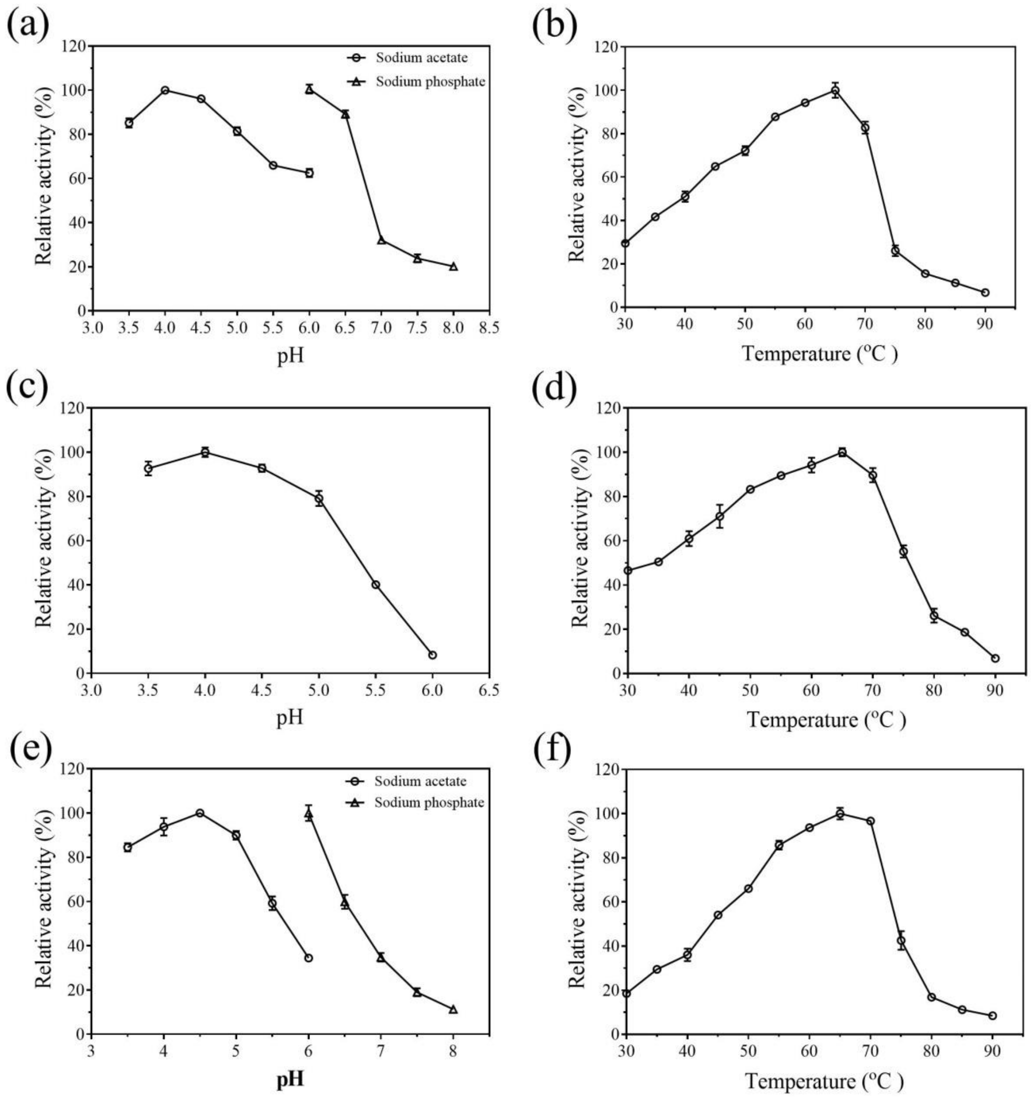

Figure **S4** Optimal pHs or temperatures of the recombinant enzymes on different substrates. (**a**) and (**b**), effect of pH or temperature on the activity of enzyme TtABF51A toward rye arabinoxylan (RAX); (**c**) and (**d**), effect of pH or temperature on the activity of enzyme TtABF51A toward sugar beet arabinan (SBA); (**e**) and (**f**), effect of pH or temperature on the activity of enzyme EpABF62C toward RAX. Except as indicated, enzymatic activities were assayed under 65 °C and in sodium acetate buffer with pH 4.0 (**b** and **d**) or 4.5 (**e**). Relative activities were calculated using the maximum activity as 100%. Error bars represent standard deviations from three independent experiments

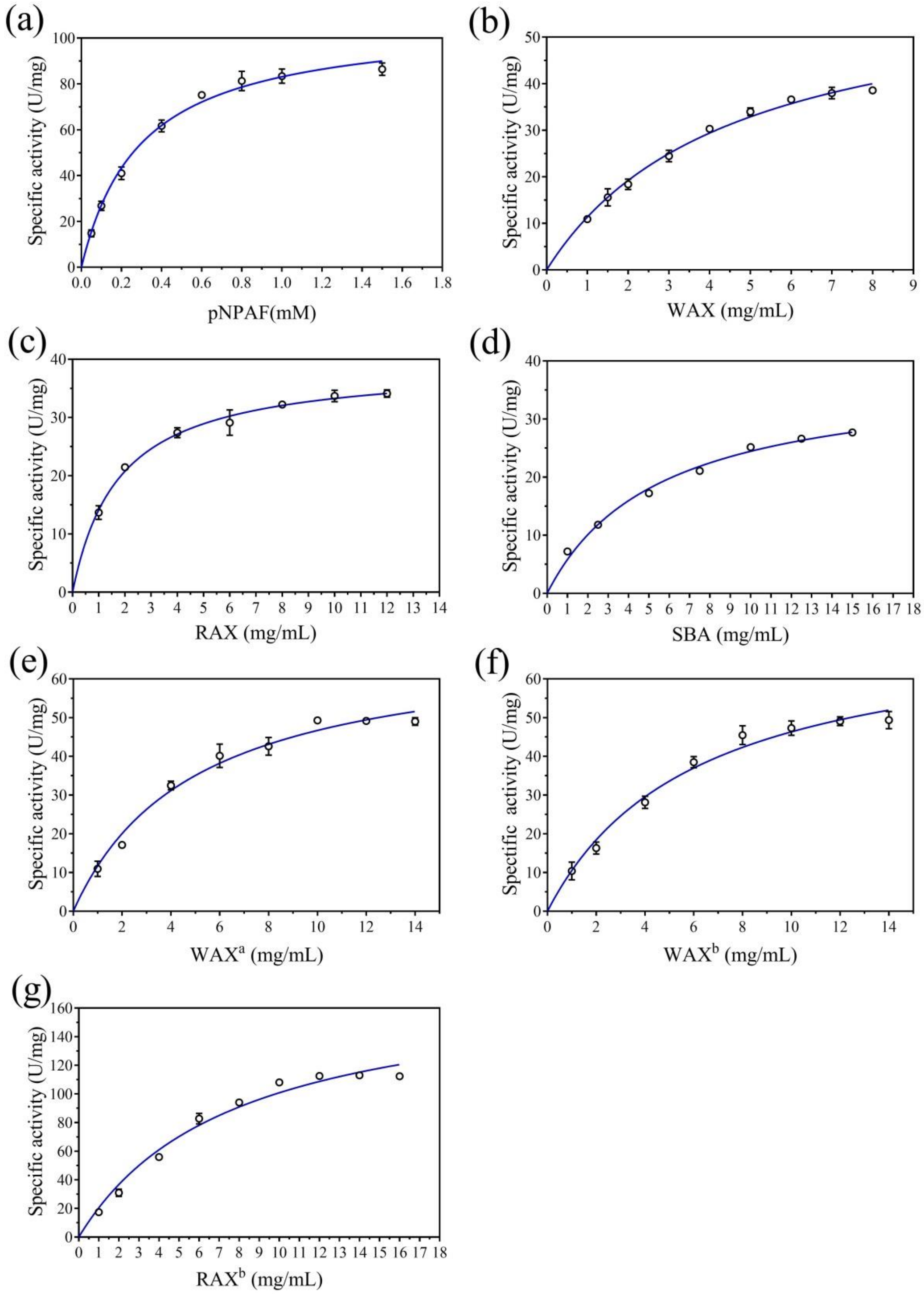

Figure **S5** Kinetic curves of recombinant enzymes toward different substrates. (**a**)-(**d**), recombinant TtABF51A; (**e**)-(**f**), recombinant EpABF62C; Enzymatic activities were detected under the optimized conditions. Kinetic curve was generated by GraphPad Prism 7.04 software using nonlinear regression. pNPAF, 4-nitrophenyl-α-l-arabinofuranoside; WAX, wheat arabinoxylan with low viscosity; RAX, rye

arabinoxylan with high viscosity; SBA, sugar beet arabinan. [a] the enzyme without Ca^+; [b] the enzyme was treated with $CaCl_2$ (2 mM). Error bars represent standard deviations from three independent assays.

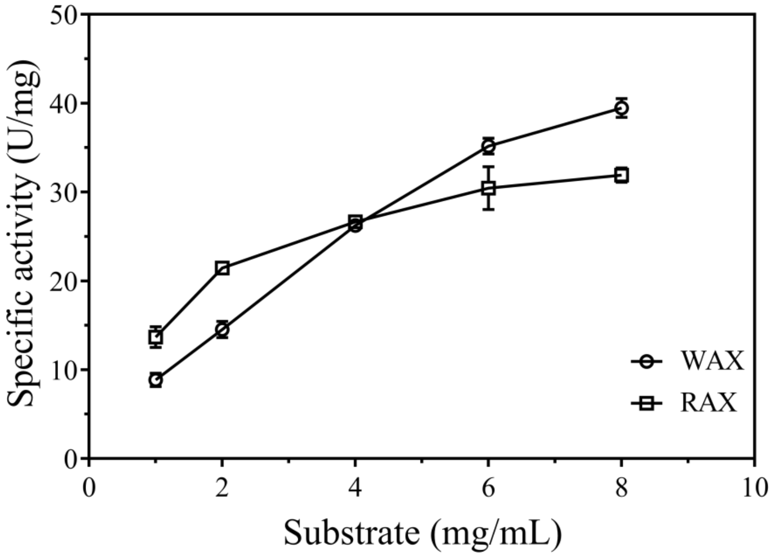

Figure **S6** Comparison of activity of enzyme TtABF51A on wheat arabinoxylan (WAX) and rye arabinoxylan (RAX) with different concentration. Enzymatic activity was measured under the standard conditions. Error bars represent standard deviations from three independent assays.

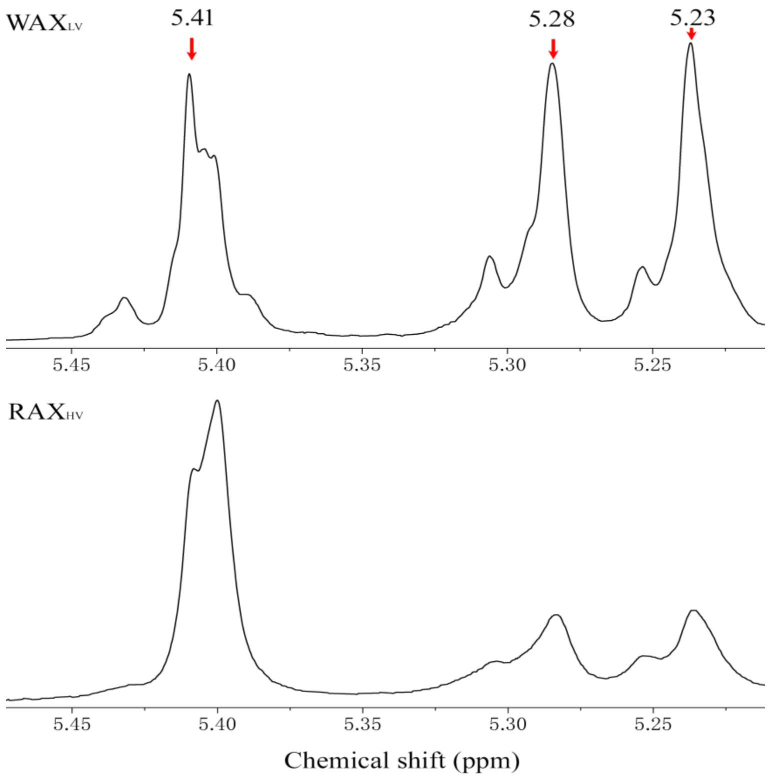

Figure **S7** 1H NMR analysis of low viscosity wheat arabinoxylan (WAX) and high viscosity rye arabinoxylan (RAX). The peak signals with chemical shifts at 5.41 ppm, 5.28 ppm and 5.23 ppm represent the mono-substituted α-1,3-L-Ara*f*, di-substituted α-1,3-L-Ara*f* and di-substituted α-1,2-L-Ara*f* in the substrates, respectively. By measurement of peak integrals, the relative intensities of mono-substituted α-1,3-L-Ara*f*, di-substituted α-1,3-L-Ara*f* and di-substituted α-1,2-L-Ara*f* is 34%, 33% and 33% on WAX, or 66%, 17% and 17% on RAX, respectively.

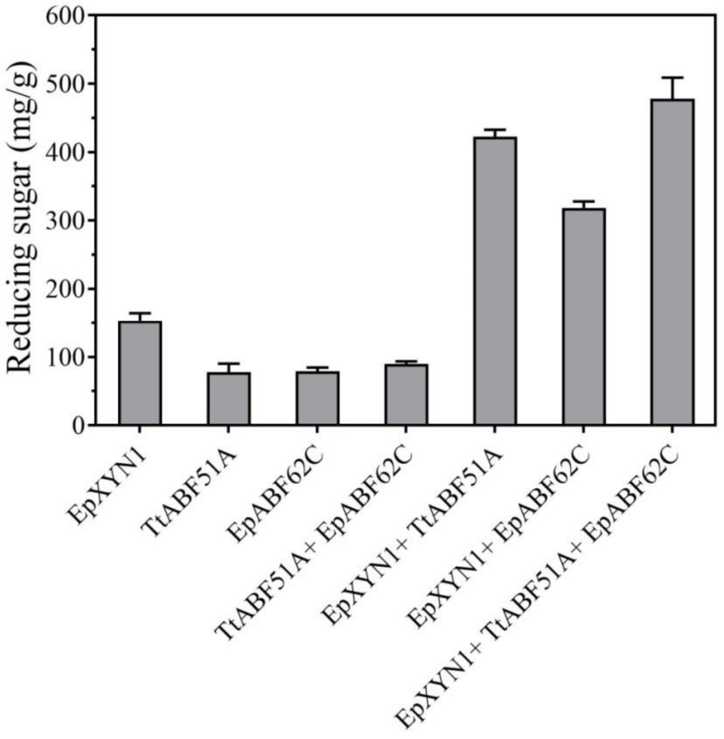

Figure **S8** Synergistic degradation of wheat arabinoxylan by xylanase with arabinofuranosidases. In 200 μL of sodium acetate buffer (50 mM, pH 4.5), 0.5 mg of wheat arabinoxylan (low viscosity) was mixed with xylanase (EpXYN1) and/or arabinofuranosidases (TtABF51A or EpABF62C). The dosage of each enzyme was 0.5 μg per reaction, and the same amount of bovine serum albumin was used as a control. Total content of reducing sugar was measured by the Somogyi-Nelson method, and calculated according to xylose standard curve.
